# Early Induction of Bedside Pneumoperitoneum in the Management of Residual Pleural Space and Air Leaks After Pulmonary Resection

**DOI:** 10.1007/s00268-020-05813-7

**Published:** 2020-10-15

**Authors:** Alessandra Pecoraro, Giovanni Maria Garbarino, Valentina Peritore, Matteo Tiracorrendo, Claudio Andreetti, Mohsen Ibrahim, Erino Angelo Rendina, Paolo Mercantini

**Affiliations:** Department of Medical Surgical Science and Translational Medicine, Sapienza University of Rome, Sant’Andrea Hospital, Via di Grottarossa, 1035-39, 00189 Rome, Italy

## Abstract

**Background:**

The pneumoperitoneum to treat prolonged air leaks or pleural space problems after pulmonary resection has been successfully used for decades. The aim of the study is to describe our experience with the early induction of therapeutic pneumoperitoneum (TP).

**Methods:**

We reviewed the data of 103 consecutive patients undergoing TP between September 2011 and September 2019. Patients were divided into two groups according to the time of the induction of TP: early application (≥72 h) and standard application (>72 h).

**Results:**

In total, 52 early TP and 51 standard TP were analyzed. The median time of TP induction was 2 (1–3) versus 8 (5–11) postoperative days (POD) (*p* < 0.001). The time for obliteration of the residual pleural space (7 vs.9 days, *p* = 0.805) and the time of resolution of the air leaks (14 vs. 16 days, *p* = 0.663) didn’t differ between the two groups, but a favorable trend was observed in the early group. The hospital stay was lower for patients undergoing early pneumoperitoneum: 9 versus 18 days (*p* < 0.001). The multivariate analysis showed that POD of induction of TP (*p* < 0.001), time of resolution of the air leak (*p* < 0.001) and Heimlich valve (*p* = 0.002) were independent variables associated with the hospital stay.

**Conclusions:**

The use of TP whenever a space problem or air leaks occur after pulmonary resections is safe and effective. Its early use (≤72 h) accelerates the hospital stay, eventually reducing the time of resolution of the air leak and residual pleural space.

## Introduction

The occurrence of prolonged air leaks and residual pleural space following lung resections is a well-known complication that thoracic surgeons strive to avoid as it causes significant morbidity [1–5].

Despite the fact that improvements in surgical techniques have contributed to reduce the incidence of such complications, residual pleural space and air leaks are still reported to occur in up to 40% of cases. Known risk factors for their occurrence include certain types of underlying lung disease, lung compliance and advance age. Therapeutic pneumoperitoneum (TP) has been frequently reported for the resolution of pleural space problems in the past two decades [6–8]. In particular, it has been successfully used to manage short-term space problems associated or not with air leaks after lung resection [9].

Although its use has been advocated to treat prolonged air leaks causing ‘basilar’ pneumothorax, our previous reports described our experience in the application of pneumoperitoneum to manage both apical and basal pleural space problems associated with air leaks [1, 9].

The aim of the study is to analyze the results of the early induction of TP, in the management of residual pleural space and air leaks developed in patients undergoing pulmonary resection.

## Material and methods

This was a retrospective observational study including all consecutive patients undergoing pulmonary resection and developing moderate to severe air leaks and postoperative residual pleural space at the Thoracic Surgery Unit of Sant’Andrea University Hospital in Rome, between September 2011 and December 2019.

The surgical procedures were classified as follows: segmentectomy, standard lobectomy, sleeve lobectomy and bilobectomy were defined as major surgeries; atypical wedge resection, bullectomy, apicoectomy, pulmonary decortication, pleural drainage of pneumothorax and excision of mediastinal lymphadenopathy were defined as minor surgeries.

The air leaks were classified as follows: (a) ‘mild’ air leaks: countable bubbles; (b) ‘moderate’ air leaks: a stream of bubbles; (c) ‘severe’ air leaks: coalescent bubbles.

Indications for the application of therapeutic pneumoperitoneum were defined by the presence of moderate to severe air leaks and by the presence of residual pleural space larger than 3 cm at chest X-ray, usually performed on the first and third postoperative day (POD), eventually associated with air leaks.

**Fig. 1:**
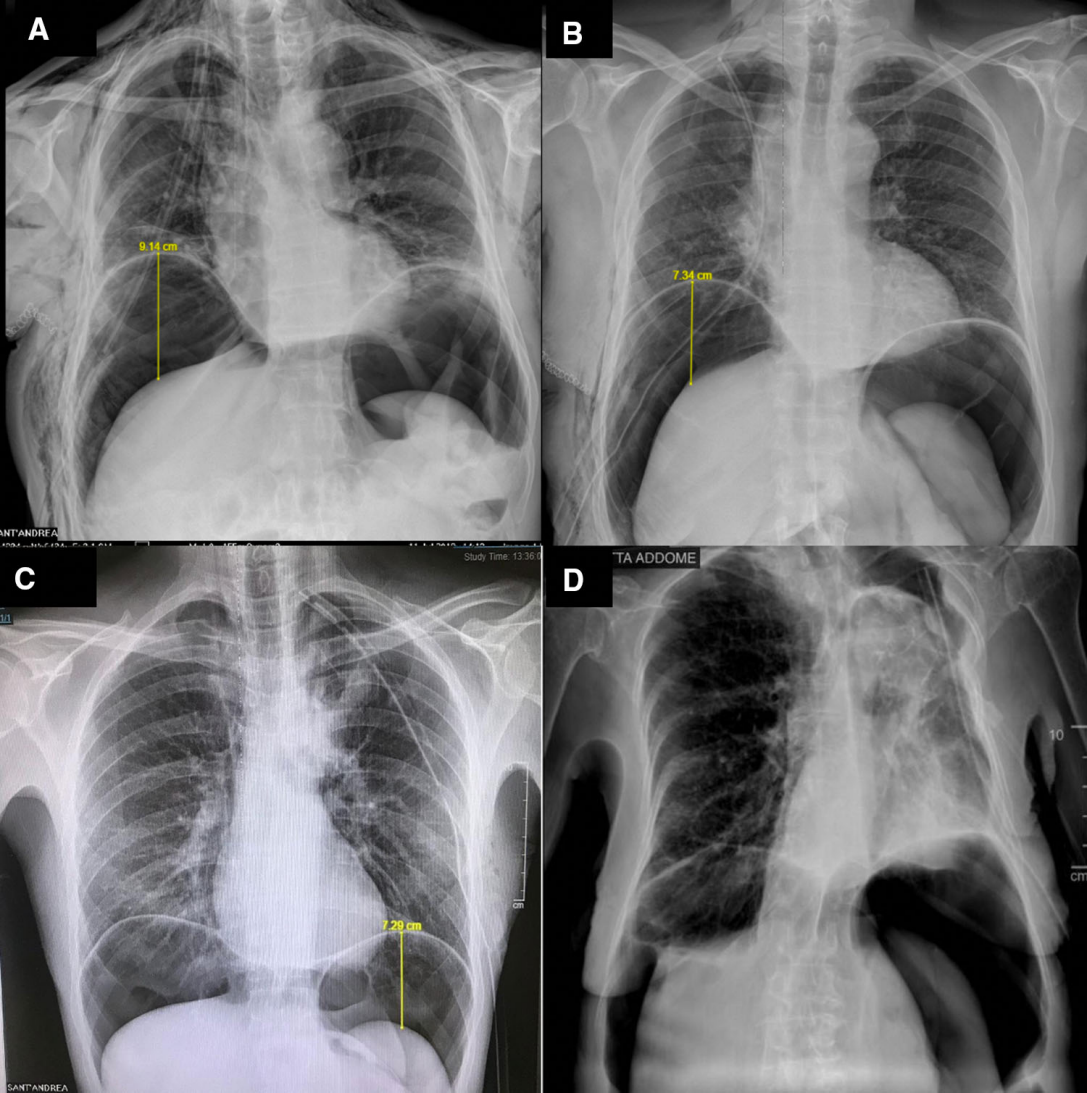
**a, b** Chest X-Ray 1 h after instillation of pneumoperitoneum for right pleural space; **c, d** chest X-Ray 1 h after instillation of pneumoperitoneum for left pleural space

After the induction of the TP, a chest and abdomen radiography was performed immediately and on the following day in order to assess the obliteration of the residual pleural space and to evaluate in centimeter the lifting of the diaphragm from the liver or the spleen, according to the side of the thoracic procedure (Fig. 1).

The chest tubes were removed 24 h after air leak stopped, documented by the disappearance of the bubbles in the drainage, and patients were discharged.

Patients with residual pleural space without air leak were discharged with Heimlich chest drainage valve. They usually underwent a chest X-ray after 72 h to evaluate the resolution or persistence of the pleural space and the eventual removal of the chest tubes.

A three-month follow-up was conducted in all the patients to exclude recurrence of parenchymal air leaks or residual pleural space.

Data were retrospectively reviewed from prospectively maintained database. Data included demographic variables, operative data, postoperative clinical and radiological data, and short-term outcomes.

Patients were divided into two groups according to the timing of postoperative therapeutic pneumoperitoneum: early induction (≤72 h) and standard induction (>72 h).

This study was conducted in accordance with the Declaration of Helsinki and its later amendments. A formal Institutional Review Board approval was not required because of the non-interventional retrospective design; however, all patients signed an informed consent for the treatment and the analysis of data for scientific purpose before any surgical procedures.

## Surgical technique

**Fig. 2:**
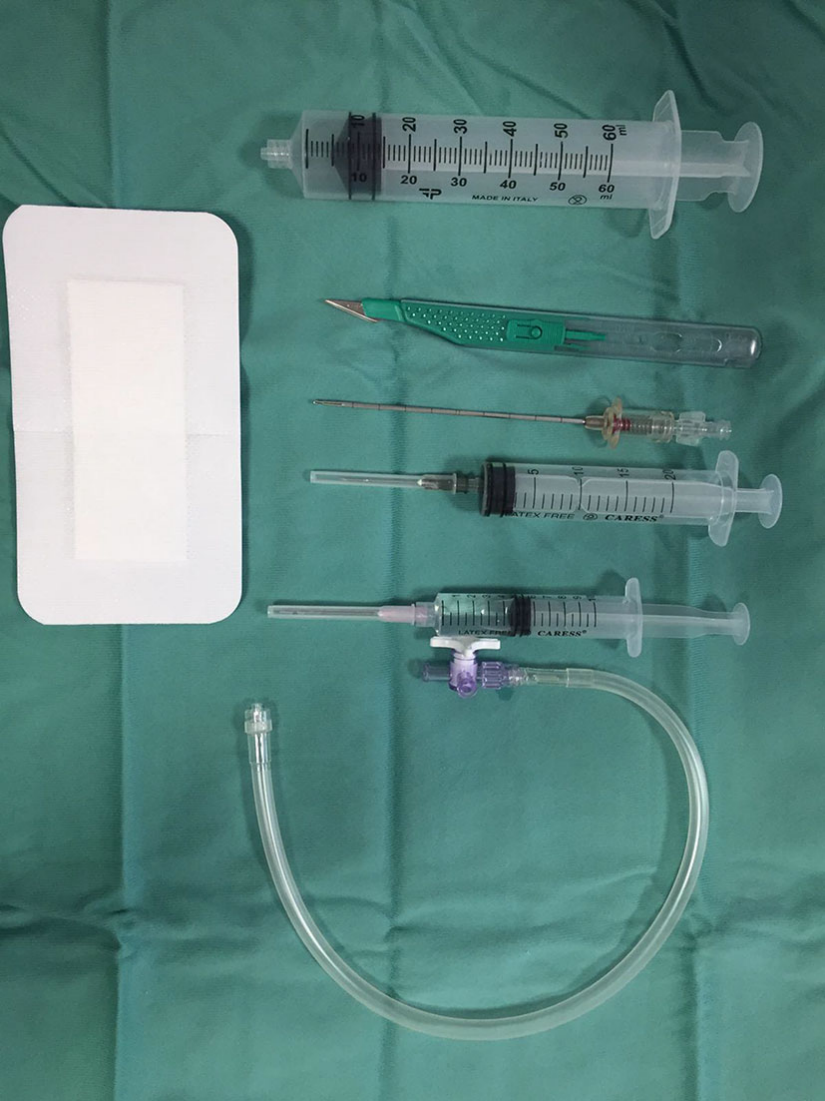
Instruments for bedside pneumoperitoneum induction

At the beginning of our experience, we performed the pneumoperitoneum in the operative room under local anesthesia and sedation. However, in the last five years the procedure was made at the bedside in local anesthesia. No Prophylactic antibiotic therapy was administered in any patients. After local infiltration in the periumbilical area, with a solution of lidocaine 2%, we use the Veress needle to inject at least 2500 mL of air in the peritoneal cavity with a 60-mL Luer Lock syringe (Fig. 2). In patients with previous abdominal surgery, we prefer use Palmer point in order to avoid intraperitoneal adhesions and intraperitoneal organ lesions. A multiparameter patient monitoring system was applied during the all procedure time.

## Statistical analysis

Continuous data were expressed as the median and interquartile range (IQR 25%-75%) because of their distribution that was assessed through the Shapiro–Wilks test. Unpaired Student t test was used to compare differences in continuous parametric variables and the Mann–Whitney U test for continuous nonparametric variables. Numbers and percentages were used for reporting categorical variables, and the Chi-square test or Fisher’s exact test with or without Yates correction was used for comparisons. To predict which variables could influence the hospital stay, a multivariable linear regression model was performed including correlate predictive factors (*p* < 0.20) at the univariable analysis and clinically relevant variables. Significance was defined as a *p* value of less than 0.05. The statistical analysis was performed using the SPSS version 25.0 (SPSS, Inc., Chicago, IL).

## Results

One hundred and three (103) patients were treated by therapeutic pneumoperitoneum and were enrolled in this study. The demographics characteristics are summarized in Table 1.

**Table 1.** Demographics characteristics of patients undergoing therapeutic pneumoperitoneum

	Total *N* = 103	Early Group (*N* = 52)	Standard Group (*N* = 51)	*P* value
Age, median (IQR 25%-75%)	68 (59–74)	65 (55–75)	70 (61–74)	0.183
Sex				
Male, *n* (%) Female *n* (%)	72 (69.9%) 31 (30.1)	37 (71.2%) 15 (28.8%)	35 (68.6%) 16 (31.4%)	0.780
Emphysema, *n*(%)	12 (11.7%)	7 (13.5%)	5 (9.8%)	0.760
FEV1 %, median (IQR 25%–75%)	83 (66–95)	85 (68–95)	81 (63–96)	0.687
ppoFEV1%, median (IQR 25%–75%)	66 (47–77)	67 (50–78)	62 (43–77)	0.440
Type of surgery				
Major, *n* (%) Minor, *n* (%)	84 (81.6%) 19 (18.4%)	39 (75.0%) 13 (25.0%)	45 (88.2%) 6 (11.8%)	0.140
Side of surgery				
Right, *n* (%) Left, *n* (%)	75 (73.0%) 28 (27.1%)	37 (71.2%) 15 (28.8%)	38 (74.5%) 13 (25.4%)	0.836
Resected lobe				
Superior, *n* (%) Inferior, *n* (%) NA, n (%)	58 (56.3%) 37 (35.9%) 8 (7.8%)	30 (57.7%) 18 (34.6%) 4 (9.3%)	28 (54.9%) 19 (37.3%) 4 (7.7%)	0.958
Air leak grading				
Mild, *n* (%) Moderate, *n* (%) Severe, n (%)	0 (0.0%) 39 (38.6%) 62 (61.4%)	0 (0.0%) 21 (41.2%) 30 (58.8%)	0 (0.0%) 18 (36.0%) 32 (64.0%)	0.593

The patients with emphysema had a higher incidence of severe air leak (5.1% vs 16.1%, *p* = 0.122), as well as patients with lower FEV1: 90 (72–99)% vs. 80 (63–90)%, *p* = 0.012 and lower ppoFEV1: 71 (52–85)% vs. 62 (45–74)%, = 0.024.

Residual pleural space and air leaks mostly occurred after major procedures. In particular, 4 (3.8%) segmentectomies, 66 (64.1%) standard lobectomies, 4 (3.8%) sleeve lobectomies, 10 (9.7%) bilobectomies, whereas only 19 (18.4%) patients underwent therapeutic pneumoperitoneum after minor surgeries.

Three patients underwent previous abdominal surgery: one radical prostatectomy, one left colectomy and one bowel resection for intestinal obstruction.

The first 35 (33.9%) procedures were performed in the operative room under sedation, as previously described [1], whereas the last 68 (66.1%) patients were treated bedside with local anesthesia.

No patients developed severe complications during and after the procedures. Minor complications included: 2 (1.9%) mild fevers, 3 (2.9%) moderate abdominal pain and dyspepsia and 1 (0.9%) atrial fibrillation. Twelve (11.7%) patients complained of shoulder referring pain, but it always disappeared with the common administration of postoperative analgesics drugs.

The postoperative characteristics of patients undergoing TP are summarized in Table 2.

**Table 2.** Postoperative characteristics of patients undergoing therapeutic pneumoperitoneum

	Total*N* = 103	Early group(*N* = 52)	Standard group(*N* = 51)	*P* value
POD of pneumoperitoneum, median (IQR 25%–75%)	3 (2–8)	2 (1–3)	8 (5–11)	<0.001
Diaphragm lifting, median (IQR 25%–75%)	7 (4–8)	7 (4–9)	7 (4–8)	0.414
Redo pneumoperitoneum, *n* (%)	6 (5.8%)	2 (3.7%)	4 (8.3%)	0.374
POD of redo pneumoperitoneum, median (IQR 25%–75%)	17 (6–32)	20 (4–n/a)	17 (8–28)	1.000
Diaphragm lifting of redo pneumoperitoneum, median (IQR 25%–75%)	7 (3–10)	8.5 (6–n/a)	6 (3–10)	0.533
Time for obliteration of pleural space, median (IQR 25%–75%)	9 (4–15)	7 (4–15)	9 (4–15)	0.805
Time for resolution of the air leak, median (IQR 25%–75%)	14 (9–22)	14 (10–20)	16 (8–27)	0.663
Hospital stay after TP, median (IQR 25%–75%)	7 (4–13)	8 (4–12)	7 (4–15)	0.534
Hospital stay, median (IQR 25%–75%)	13 (8–21)	9 (7–14)	18 (12–26)	<0.001

In 6 patients, it was necessary to refill the pneumoperitoneum with almost additional 1000 cc of air because of persistent residual pleural space. Among these patients, three underwent a left superior lobectomy, one a right superior lobectomy, one a right bilobectomy and one a left atypical resection.

No recurrence of air leak was observed during a follow-up of 3 months after hospital discharge, whereas six patients (6.8%) didn’t show a complete obliteration of the residual pleural space**.** Among these patients, two underwent a right superior lobectomy, one a left inferior lobectomy, one a left superior lobectomy, one a left atypical resection and the last patient underwent pleural drainage.

According to univariable linear regression analysis, emphysema (*p* = 0.154), air leak grading (*p* = 0.024), POD of induction of TP (*p* < 0.001), time for obliteration of the pleural space (*p* < 0.001), as well as the time of resolution of the air leak (*p* < 0.001), increased the odds of a longer hospital stay while a high FEV1 (*p* = 0.056) and ppoFEV1 (*p* = 0.012), the left side of pulmonary resection (*p* = 0.116) and the presence of the Heimlich valve (*p* = 0.146) decreased the odds. These variables were included in a multivariable linear model which showed that POD of induction of TP (*p* < 0.001) and the time of resolution of the air leak (*p* < 0.001) were independent variables associated with a longer hospital stay, whereas the Heimlich valve (*p* = 0.002) was an independent factor associated with a faster hospitalization (Table 3).

**Table 3.** Linear regression model for predictive factors of length of hospital stay.

	Univariable analysis		Multivariable analysis	
	OR (95% CI)	*P* value	OR (95% CI)	*P* value
Age (years)	0.01 (−0.16–0.17)	0.950		
Sex	−0.07 (−6.58–3.31)	0.514		
Emphysema	0.14 (−1.9–12.1)	0.154	0.09 (−0.89–6.14)	0.142
FEV1	−0.19 (−0.21–0.01)	0.056	−0.04 (−0.13–0.09)	0.747
ppoFEV1	−0.25 (−0.23 to −0.03)	**0.012**	0.03 (−0.09–0.12)	0.805
Type of surgery (major/minor)	0.10 (−2.96–8.71)	0.331		
Side of surgery (left/right)	−0.16 (−9.30–1.04)	0.116	−0.02 (−2.92–2.18)	0.775
Resected lobe (superior/inferior)	−0.07 (−6.75–3.17)	0.476		
Air leak grading (moderate/severe)	0.22 (0.57–8.08)	**0.024**	0.02 (−3,–2.43)	0.810
POD of pneumoperitoneum	0.49 (0.72–1.49)	**<0.001**	0.57 (0.78–1.24)	**<0.001**
Diaphragm lifting	−0.09 (−1.26–0.50)	0.393		
Heimlich valve	−0.15 (−10.8–1.62)	0.146	−0.20 (−9.32 to −2.24)	**0.002**
Time for obliteration of pleural space	0.58 (0.41–0.74)	**<0.001**	−0.05 (−0.29–0.19)	0.683
Time of resolution of the air leak	0.47 (0.26–0.57)	**<0.001**	0.57 (0.28–0.77)	**<0.001**

A subgroup analysis based on the air leak grading revealed no differences between moderate and severe air leak in terms of timing of the induction of the TP: 4 (2–8) versus 3 (2–8) days, *p* = 0.970, while the time for resolution of the air leak was longer for the severe group: 11 (7–14) versus 25 (17–30) days, *p* < 0.001.

Patients were then divided into two groups according to the time of the induction of postoperative therapeutic pneumoperitoneum: 52 (50.5%) patients underwent early (≤72 h) and 51 (49.5%) patients standard (> 72 h) pneumoperitoneum.

There were no significant differences between the two groups in terms of age, sex, side of the surgery, upper/lower resected lobe, diaphragm lifting and redo pneumoperitoneum (Table 1).

The patients treated with early TP had shorter time of obliteration of the pleural space 7 (4–15) versus 9 (4–15) days, *p* = 0.805, and time to resolution of the air leaks: 14 (9–19) versus 16 (8–27) days, *p* = 0.663; even if not statistically significant.

The median hospital stay after TP didn’t differ between the two groups: 8 (4–12) versus 7 (4–15) days, *p* = 0.534.

The only significant difference between the two groups was that patients undergoing early pneumoperitoneum had a shorter hospital stay than patients treated by standard TP: 9 (7–14) versus 18 days (*p* < 0.001).

Concerning the 3-month follow-up, among the patients who didn’t show a complete obliteration of the residual pleural space, three were treated by early TP and three with standard TP.

## Discussion

Prolonged air leak is an alarmingly common postoperative complication that amounts to 25.5% of all complications following a pulmonary resection. It is the most frequent cause of a prolonged hospital stay in patients undergoing lobectomy, especially radical upper lobectomy. Severe obstructive pulmonary disease is the major risk factor for the development of this complication [10]. Indeed, in the current study, patients with emphysema, lower FEV1 and ppoFEV1 had a higher rate of severe air leak.

The use of pneumoperitoneum to treat prolonged air leaks in patients with basal spaces after lung resection has been well known since 1999 [6]. Recently, some authors recommended its use to manage apical spaces and prolonged air leaks following lung volume reduction surgery [11].

The physiologic mechanisms working to compensate the reduction in the lung volume after pulmonary resections are: the mediastinal shifting, the elevation of homolateral hemidiaphragm and the expansion of the residual lung parenchyma. Several factors predispose to residual pleural space such as prolonged air leak including restrictive lung diseases, previous thoracic surgery and preoperative radiotherapy or chemotherapy.

Therapeutic pneumoperitoneum, allowing temporary elevation of the diaphragm, is effective in both apical and basal air spaces. Furthermore, it is easy to perform and has no long-term sequelae.

The reduction in lung volume relative to chest cavity volume determines a problem of residual pleural space. The visceral–parietal pleural apposition is caused by the introduction of air into the peritoneal cavity, which displaces the diaphragm cephalad and decreases the volume of the chest cavity. The intraperitoneal air is reabsorbed in about 7–14 days after insufflation. During this period, the pleural fusion usually occurs, leading to a gradual re-expansion of chest cavity and lung volumes [12, 13].

A space problem may come up after each kind of pulmonary resection, but it is more frequent after lobectomies or bilobectomies. Thus, Okur et al. designed a prospective randomized trial to evaluate the intraoperative pneumoperitoneum after lower lobectomy or lower bilobectomy for lung cancer or inflammatory lung diseases. This study showed that intraoperative pneumoperitoneum is a safe and simple procedure, and it allows a faster removal of chest drainage and a reduced hospital stay [7].

To the best of our knowledge, the current study is the largest series of patients treated with therapeutic postoperative pneumoperitoneum for the management of air leaks and residual pleural space after pulmonary resection. Moreover, most of the previous reports only described the use of the pneumoperitoneum to treat residual pleural space and air leaks developed after major pulmonary resections [1, 7–9]. In the present series, this therapeutic tool was also applied for the treatment of the aforesaid complications following minor surgeries such as atypical wedge resection, bullectomy, apicoectomy, pulmonary decortication, pleural drainage of pneumothorax and excision of mediastinal lymphadenopathy.

At the beginning of our experience, we started to induce pneumoperitoneum late in the postoperative course because we considered the procedure quite invasive [1, 9]. The pneumoperitoneum was induced in the operative room under local anesthesia and sedation, with the involvement of several operating room nurses and an anesthesiologist. Recently, the authors decided to induce pneumoperitoneum precociously during the hospital stay. In fact, during the last five years, almost all the patients underwent pneumoperitoneum within the third postoperative, and 22 (21.3%) patients were treated by TP in the first postoperative day. The early use of this technique can allow a faster obliteration of the residual pleural space and resolution of the air leak, likely because of increased lung mobility and ability to shift to the apex of the chest cavity in the early postoperative period. Early application of this technique, can result in shorter hospital stay, as demonstrated by our results. In fact, as shown by the multivariate analysis, the patients who underwent an earlier induction of TP and the patients discharged with Heimlich chest drainage valve had a faster hospital stay.

Our technique has evolved over time from a formal operative room procedure to a bedside one. This shifting in the operative setting lets the surgical team to promptly perform TP, without waiting for the end of the scheduled surgeries or for the availability of the anesthesiologist. This factor has encouraged us toward a greater usage of this technique and also at an earlier stage, allowing shorter hospital stay for patients and significant cost savings for our institution. In our series, we also would like to report one patient who underwent TP as an outpatient due to a persistent air leak treated with Heimlich valve drain. The patient was discharged immediately after the procedure without any complication, and 24 h after we observed complete resolution of the air leak and were able to remove the Heimlich valve drain.

This study has several limitations. It is a retrospective study over a long period of time with a relatively small sample size. However, despite these limitations, this study confirms that therapeutic pneumoperitoneum is a simple and safe technique which can be used postoperatively to reduce the chest volume and promote the resolution of the air leaks and residual pleural space.

In conclusion, on the basis of previous reports and our own experience, we believe that therapeutic pneumoperitoneum is a very useful and effective option. It has very limited costs and is able to achieve optimal pleural apposition, appropriate dead space obliteration and resolution of air leak. Its early application (≤72 h) results in a shorter hospital stay, eventually reducing the time of resolution of the air leak and residual pleural space.
